# Prospecting for viral natural enemies of the fire ant *Solenopsis invicta* in Argentina

**DOI:** 10.1371/journal.pone.0192377

**Published:** 2018-02-21

**Authors:** Steven M. Valles, Sanford D. Porter, Luis A. Calcaterra

**Affiliations:** 1 Center for Medical, Agricultural and Veterinary Entomology, USDA-ARS, Gainesville, Florida, United States of America; 2 Fundación para el Estudio de Especies Invasivas, Bolívar, B1686EFA Hurlingham, Buenos Aires, Argentina; University of British Columbia, CANADA

## Abstract

Metagenomics and next generation sequencing were employed to discover new virus natural enemies of the fire ant, *Solenopsis invicta* Buren in its native range (i.e., Formosa, Argentina) with the ultimate goal of testing and releasing new viral pathogens into U.S. *S*. *invicta* populations to provide natural, sustainable control of this ant. RNA was purified from worker ants from 182 *S*. *invicta* colonies, which was pooled into 4 groups according to location. A library was created from each group and sequenced using Illumina Miseq technology. After a series of winnowing methods to remove *S*. *invicta* genes, known *S*. *invicta* virus genes, and all other non-virus gene sequences, 61,944 unique singletons were identified with virus identity. These were assembled *de novo* yielding 171 contiguous sequences with significant identity to non-plant virus genes. Fifteen contiguous sequences exhibited very high expression rates and were detected in all four gene libraries. One contig (Contig_29) exhibited the highest expression level overall and across all four gene libraries. Random amplification of cDNA ends analyses expanded this contiguous sequence yielding a complete virus genome, which we have provisionally named Solenopsis invicta virus 5 (SINV-5). SINV-5 is a positive-sense, single-stranded RNA virus with genome characteristics consistent with insect-infecting viruses from the family *Dicistroviridae*. Moreover, the replicative genome strand of SINV-5 was detected in worker ants indicating that *S*. *invicta* serves as host for the virus. Many additional sequences were identified that are likely of viral origin. These sequences await further investigation to determine their origins and relationship with *S*. *invicta*. This study expands knowledge of the RNA virome diversity found within *S*. *invicta* populations.

## Introduction

The red imported fire ant, *Solenopsis invicta* Buren is an invasive species native to southern South America [1]. The ant was introduced into North America sometime in the 1930s [1], most likely from somewhere in Formosa Province, Argentina [2]. This ant is a very serious pest in the U.S., but generally not in its native range; although it is one of the most ecologically dominant ant species in northeastern Argentina [3, 4]. Damage and control efforts cost an estimated $6 billion annually in the U.S. [5]. Population studies on the two continents have shown that fire ant populations are 5–10 times greater in infested areas within the U.S. [6, 7]. These inter-continental disparities support the supposition that *S*. *invicta* likely escaped its natural enemies during U.S. founding events. Indeed, direct evaluations have shown a paucity of natural enemies in founding populations of *S*. *invicta* [8].

In the U.S., early eradication efforts were attempted [1], but eventually gave way to the implementation of quarantine [9] to limit the spread of the ant. Concomitantly, research focus shifted from eradication to the discovery, characterization, and release of natural enemies of *S*. *invicta* with the intention of providing sustainable control in the U.S. This effort led to the discovery of many pathogens and parasites of fire ants in their native and introduced ranges [10], some of which have been utilized and released as natural control agents against invasive fire ants in the U.S. [11–13]. Still, there remains a large discrepancy in both the abundance and the number of natural enemies found between populations of *S*. *invicta* in South and North America [14, 15] warranting continued efforts to identify new pathogens for use in providing natural control.

Despite the known usefulness of viruses to control insect pests [16], viruses have been only recently investigated for use against ants [11, 17]. Indeed, the first ant viruses discovered and characterized were from *S*. *invicta* [14]. To date, four RNA viruses and one DNA virus have been discovered from *S*. *invicta*. The RNA viruses include Solenopsis invicta virus 1 [18], Solenopsis invicta virus 2 [19], Solenopsis invicta virus 3 [20], and Solenopsis invicta virus 4 [21]. All of these viruses are present in both U.S. and Argentine populations of *S*. *invicta*. *Solenopsis invicta* queens infected with Solenopsis invicta virus 1 (SINV-1) have lower body weights that reduce the probability of successful colony founding [22]. Solenopsis invicta virus 2 (SINV-2) infections are associated with significant reductions in queen fecundity and other detrimental fitness effects including longer claustral periods and slower growth of incipient colonies [22]. Solenopsis invicta virus 3 (SINV-3) also reduces queen fecundity [23] and alters the feeding behavior exhibited by the worker caste, which results in colony starvation [24]. The impacts of Solenopsis invicta virus 4 (SINV-4) and the sole DNA virus, Solenopsis invicta densovirus (SiDNV), have not been established [25].

The objective of this research was to discover new virus natural enemies of *S*. *invicta* from the native range (i.e., Formosa, Argentina), with the ultimate goal of their release into introduced U.S. populations as self-sustaining biocontrol agents. Metagenomics and next generation sequencing were employed to achieve this objective and resulted in the discovery of one new virus and multiple high-probability target sequences of likely viral origin providing future leads to pursue.

## Materials and methods

### *Solenopsis invicta* collections

**Fig 1:**
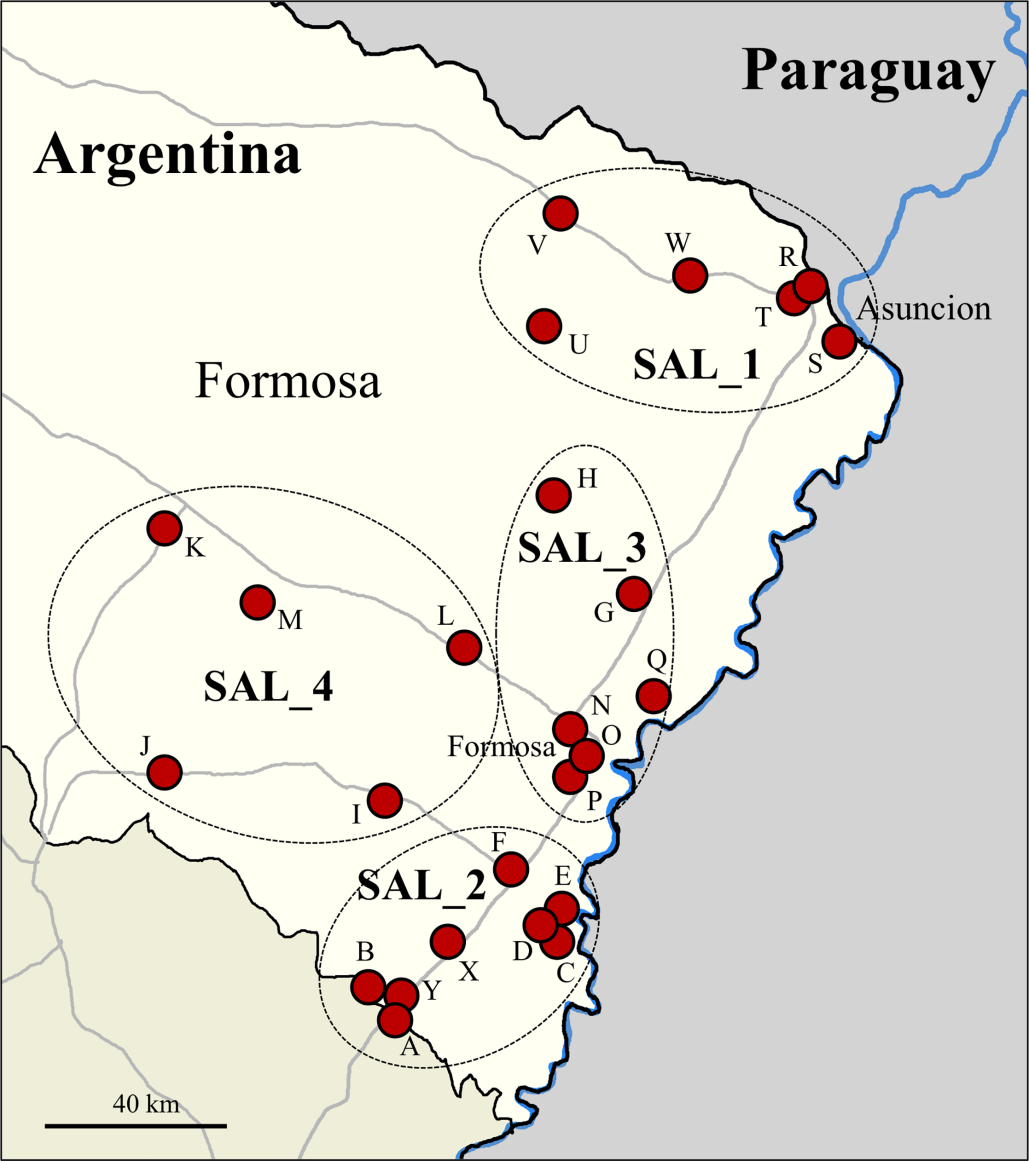
Map of collection locations (capital letters) in Formosa Province, Argentina. Dashed ovals show how locations were grouped for RNA library preparation. We sampled 2–9 colonies at each site. See Table 1 for colony numbers, GPS coordinates, and habitat descriptions.

Samples of workers, brood, and sexuals were obtained from a total of 182 *S*. *invicta* colonies collected at 25 locations in the eastern portion of the Province of Formosa, Argentina (Fig 1). Collections were limited to the Province of Formosa by design because *S*. *invicta* in the U.S. has been shown to originate from this region [2]. Pathogen-matching based on genetic relatedness can have a profound influence on the ability of pathogens to infect a host [26]. Collection details, including specific location, date, and habitat are summarized in Table 1. After removal from the nest, live ants were held in 50 ml centrifuge tubes with a screened top and transported to the USDA quarantine facility in Gainesville, Florida, where they were placed in rearing trays and provided water and sugar water. We removed a sample of 15 workers 11–21 days after collection from each colony fragment for RNA extraction and any remaining individuals were frozen at -80 ^o^C for future evaluations. Voucher specimens have been deposited in both the USDA-ARS, Center for Medical, Agricultural and Veterinary Entomology (CMAVE), Gainesville, Florida collection and the Fundación Para el Estudio de Especies Invasivas (FuEDEI), Hurlingham, Buenos Aires, Argentina collection.

**Table 1:** Summary of collection information for *Solenopsis invicta* colonies used in RNA library preparation. Library designation represents South American Library (SAL).

Site	Date	Latitude	Longitude	Habitat	Number of colonies	Library designation
R	23-ix-14	-25.2875	-57.7054	Dike along river	7	SAL_1
S	22-ix-14	-25.3598	-57.6631	Dike	5	SAL_1
T	22-ix-14	-25.2948	-57.7445	Road right of way	8	SAL_1
U	24-ix-14	-25.3553	-58.2796	Low grass	9	SAL_1
V	24-ix-14	-25.1432	-58.2314	Roadside	9	SAL_1
W	24-ix-14	-25.2508	-57.9907	Roadside	8	SAL_1
A	16-ix-14	-26.6609	-58.6297	River bank, Costanera park	8	SAL_2
B	16-ix-14	-26.6222	-58.6653	Semi urban	9	SAL_2
C	17-ix-14	-26.5173	-58.2818	Field adjacent to road	9	SAL_2
D	17-ix-14	-26.4806	-58.2737	Marshy road area	7	SAL_2
E	17-ix-14	-26.4795	-58.3071	City park, mowed grass	9	SAL_2
F	17-ix-14	-26.3944	-58.3442	Gas station	7	SAL_2
X	25-ix-14	-26.5412	-58.5081	Roadside	1	SAL_2
Y	25-ix-14	-26.6474	-58.6236	Roadside	3	SAL_2
G	18-ix-14	-25.8786	-58.0872	Abandoned parking area and periphery	9	SAL_3
H	18-ix-14	-25.6733	-58.2561	Grassy roadside	6	SAL_3
N	20-ix-14	-26.1182	-58.2241	Urban, highly disturbed	5	SAL_3
O	22-ix-14	-26.1975	-58.2144	Adjacent to gas station	7	SAL_3
P	22-ix-14	-26.2281	-58.2405	Abandoned picnic area	9	SAL_3
Q	22-ix-14	-26.0416	-58.0589	Rural road side	8	SAL_3
I	19-ix-14	-26.2391	-58.6303	Rough grassy area	9	SAL_4
J	19-ix-14	-26.205	-59.0708	City park	8	SAL_4
K	19-ix-14	-25.7411	-59.1021	Mowed road edge, west side	9	SAL_4
L	20-ix-14	-25.9357	-58.5124	Road edge	5	SAL_4
M	20-ix-14	-25.8637	-58.8858	Residential area	8	SAL_4

### RNA preparation

Total RNA was extracted from a pooled group of 15 worker ants from each colony fragment using the Trizol method followed by the PureLink RNA Mini Purification Kit according to the manufacturer’s instructions (Thermo Fisher Scientific, Waltham, MA). RNA quality of each preparation was assessed by microfluidic analysis on an Agilent 2100 Bioanalyzer (Agilent, Cary, NC) using the RNA 6000 Nano kit according to the manufacturer’s instructions. RNA samples were pooled from ant colonies into four groups according geographic region (Table 1; Fig 1). The four groups included South American Library _1 (SAL_1), collected north of 25.4^o^ latitude; SAL_2, collected south of 26.3^o^ latitude; SAL_3, collected east of 58.3^o^ longitude; and SAL_4, collected west of 58.5^o^ longitude. The total number of colonies pooled in each group was 46 (SAL_1), 53 (SAL_2), 44 (SAL_3), and 39 (SAL_4). Total RNA (10 μg per group) was submitted to GE Healthcare (Los Angeles, CA) for mRNA purification, library preparation, and Illumina RNA sequencing (MiSeq).

### Library preparation and sequencing

Total RNA (200 ng) purified from each of the four pooled groups of worker ants (SAL_1, SAL_2, SAL_3, and SAL_4) was used for mRNA purification with the Illumina TruSeq Stranded mRNA Library Preparation Kit (Catalog # RS-122-2101). The low sample protocol was followed according to the manufacturer’s instructions. The RNA fragmentation step was omitted to maximize library insert length. Rather than fragmenting the RNA, the sealed plate was incubated at 80°C for 2 minutes to elute the primed mRNA from the RNA purification beads. This omission resulted in RNA fragmentation with an average final library size of 467 bp. Library sizes were determined empirically by microfluidic analysis on an Agilent 2100 Bioanalyzer and quantified using the Quant-iT dsDNA kit with broad range standards (ThermoFisher Scientific, Q-33130). Samples were pooled together at equimolar quantities and sequenced twice using the Illumina MiSeq (2x300) cycle kit with version 3 chemistry. Using the Illumina indices, the data were demultiplexed and the runs combined to assign the data to individual samples. All other procedures were followed according to the manufacturer’s instructions.

### Bioinformatics analysis

Sequences were aligned to the *Solenopsis invicta* reference genome downloaded from http://www.ncbi.nlm.nih.gov/Traces/wgs/?val=AEAQ01#contigs using the Burrows-Wheeler Aligner (bwa-0.7.5a), a software package for mapping low-divergent sequences against a large reference genome [27]. The *S*. *invicta* unmapped reads were selected and converted to FASTA format using NextGENe-2.3.4 (SoftGenetics, State College, PA). Each read was then filtered and retained if the median score was ≥ 20 and base number ≥ 25. Unmapped and filtered individual MiSeq sequences were analyzed using BLASTX [28] against the curated Swiss Protein database (http://www.uniProt.org; download date 11/14/2014). Sequences returning an expectation score less than 10^−5^ were tabulated.

Based on the BLASTX results, each sequence was annotated and assorted taxonomically. The sequences were binned into the following groups: Animal, Plant, Fungi, Bacteria, Archaea, Phage, and Non-phage virus. Also at this stage, sequences exhibiting identity to Enterobacteria phage phiX174, an internal control for Illumina processing [29] were removed and not considered in subsequent analyses.

### Virus sequences

Non-phage virus sequences from each library identified from the BLASTX analysis were assembled using the CAP3 algorithm [30] in the Vector NTI ContigExpress program (Invitrogen, Carlsbad, CA). Sequences from phage were not assembled because they infect bacteria and would not be expected to infect fire ant cells. Contiguous sequences (contigs) and remaining singletons were matched to the genomes of known fire ant viruses (Solenopsis invicta virus 1 [SINV-1, GCF_000854925.1], SINV-2 (GCF_000870805.1), SINV-3 (GCF_000881215.1), SINV-4 (MF_041808.1), and SiDNV (GCF_000912895.1)). Those sequences matching known fire ant viruses (≥ 95% identity) were binned according to virus species and excluded from further analysis. Virus unmatched sequences/contigs were re-analyzed by BLASTX and sequences returning an expectation score of less than 10^−5^ were tabulated.

### Data availability

Raw sequence data from each library were deposited into the GenBank database as a Sequence Read Archive under accession number, SRP113235 (Bioproject PRJNA394996). Assembled sequences with viral identity (Table 2) have been deposited at DDBJ/EMBL/GenBank as a Transcriptome Shotgun Assembly project under the accession GFUG00000000. The version described in this paper is the first version, GFUG01000000. The SINV-5 annotated genome was deposited in GenBank under accession number MF593921.

**Table 2:** Contiguous sequences (Contigs) with significant viral identity by BLASTX analysis of the GenBank database and considered high likelihood viral prospects from RNA libraries created from *Solenopsis invicta* worker ants. Contigs were first sorted in descending order based on the number of the sequences comprising it, followed by the libraries represented.

Designation	Sequences comprising contig	Size (nt)	e-score	Query coverage:identity (%)	Sequence identity with	Number of singletons in				Genome	Virus family
						SAL_1	SAL_2	SAL_3	SAL_4		
Contig_29 *(SINV-5)*	21294	9030	0	57: 67	Israeli acute paralysis virus	274	10312	4312	6396	ssRNA	Dicistroviridae
Contig_66	13980	2626	2E^-154^	76: 39	Aphid lethal paralysis virus	3823	1704	5241	3212	ssRNA	Dicistroviridae
Contig_30	6197	1869	1E^-139^	96: 40	Aphid lethal paralysis virus	2165	779	1833	1420	ssRNA	Dicistroviridae
Contig_16	2104	2371	0	83:48	Solenopsis invicta virus 1	2	133	1815	154	ssRNA	Dicistroviridae
Contig_70	641	1068	6E^-64^	82: 43	Alber virus	117	180	141	203	RNA	Unclassified
Contig_58	607	1453	1E^-67^	88: 34	Nasonia vitripennis virus	18	311	37	241	RNA	Unclassified
Contig_13	510	1568	2E^-102^	87: 41	Aphid lethal paralysis virus	206	98	7	199	ssRNA	Dicistroviridae
Contig_21	486	3211	0	85: 61	Acute bee paralysis virus	108	51	9	318	ssRNA	Dicistroviridae
Contig_83	428	2623	0	97: 62	Mosinovirus	90	58	110	170	RNA	Unclassified
Contig_55	202	2639	3E^-147^	91: 35	Kashmir bee virus	12	153	20	17	ssRNA	Dicistroviridae
Contig_82	114	1203	0	82: 94	Acute bee paralysis virus	16	7	4	87	ssRNA	Dicistroviridae
Contig_15	100	1042	0	92: 83	Hubei orthoptera virus 1	28	14	3	55	RNA	Unclassified
Contig_27	69	680	6E^-135^	91: 97	Acute bee paralysis virus	1	6	2	60	ssRNA	Dicistroviridae
Contig_17	58	796	4E^-162^	99: 91	Acute bee paralysis virus	15	7	5	31	ssRNA	Dicistroviridae
Contig_80	55	1173	0	98: 79	Drosophila C virus	44	4	3	4	ssRNA	Dicistroviridae
Contig_75	36	589	6E^-26^	83: 39	Hubei picorna-like virus 46	8	13	15	0	RNA	Unclassified
Contig_47	734	1045	7E^-82^	89: 44	Solenopsis invicta virus 2	0	667	6	61	ssRNA	Unclassified
Contig_78	216	702	7E^-111^	99: 85	Israeli acute paralysis virus	153	42	21	0	ssRNA	Dicistroviridae
Contig_57	146	1028	5E^-116^	93: 57	Wuhan insect virus 11	0	93	28	25	RNA	Unclassified
Contig_18	44	664	2E^-84^	97: 60	Hubei Orthoptera virus 1	10	2	0	32	RNA	Unclassified
Contig_88	44	1500	0	99: 87	Rhopalosiphum padi virus	12	0	7	25	ssRNA	Dicistroviridae
Contig_53	39	522	1E^-50^	98: 52	Wuhan insect virus 11	0	27	3	9	RNA	Unclassified
Contig_9	9	471	3E^-68^	87: 95	Acute bee paralysis virus	3	0	3	3	ssRNA	Dicistroviridae
Contig_85	8	542	2E^-53^	91: 56	Solenopsis invicta virus 1	4	1	3	0	ssRNA	Dicistroviridae
Contig_51	321	1465	0	99: 76	Nodamura virus	0	274	0	47	ssRNA	Nodaviridae
Contig_49	182	1269	0	99: 95	Aphid lethal paralysis virus	0	153	29	0	ssRNA	Dicistroviridae
Contig_12	95	438	2E^-79^	99: 77	Big Sioux River virus	49	0	0	46	ssRNA	Unclassified
Contig_50	69	768	9E^-68^	99: 46	Israeli acute paralysis virus	0	68	0	1	ssRNA	Dicistroviridae
Contig_60	69	926	0	99: 93	Aphid lethal paralysis virus	0	55	14	0	ssRNA	Dicistroviridae
Contig_28	67	830	1E^-46^	97: 37	Israeli acute paralysis virus	0	0	13	54	ssRNA	Dicistroviridae
Contig_52	66	356	1E^-35^	99: 94	Shuangao insect virus 8	0	20	0	46	RNA	Unclassified
Contig_25	58	869	8E^-10^	37: 40	Hubei picorna-like virus 50	0	0	20	38	RNA	Unclassified
Contig_141_3101	3101	2351	0	97: 55	Solenopsis invicta virus 1	0	0	0	3101	ssRNA	Dicistroviridae
Contig_19	1075	1080	2E^-159^	89: 71	Solenopsis invicta virus 1	0	0	0	1075	ssRNA	Dicistroviridae
Contig_19_480	480	623	8E^-114^	99: 88	Solenopsis invicta virus 1	0	0	480	0	ssRNA	Dicistroviridae
Contig_110_361	361	1214	0	99: 82	Kashmir bee virus	0	0	0	361	ssRNA	Dicistroviridae
Contig_81_84	84	1103	6E^-161^	99: 67	Wuhan arthropod virus 2	0	0	84	0	RNA	Unclassified
Contig_108_79	79	715	5E^-131^	98: 83	Shuangao insect virus 8	0	79	0	0	RNA	Unclassified

### Virus genome re-sequencing

Contig_29 was unquestionably a near complete virus RNA genome. Therefore, this sequence was used as template for 5' and 3' RACE to acquire the entire genome sequence. For 3' RACE, cDNA was synthesized with the GeneRacer Oligo dT primer (Invitrogen, Carlsbad, CA). PCR was subsequently conducted with the GeneRacer 3' primer and gene-specific primer, P1604 (S1 Table). For 5' RACE, cDNA was synthesized with oligonucleotide primer P1601 and PCR conducted with P1601 and the GeneRacer Abridged Anchor Primer. Amplicons generated during RACE reactions were cloned into pCR4 vector and submitted for Sanger sequencing. After acquiring the genome termini, oligonucleotide primers were designed to provide complete, overlapping coverage of Contig_29. Amplicons were cloned and sequenced by the Sanger method and the genome assembled with CAP3 in Vector NTI (Life Technologies, Carlesbad, CA).

### Preliminary RNA virus confirmation

In order to provide further evidence of whether a contig was of viral origin, or not, oligonucleotide primers (S1 Table) were designed to contigs with viral identity and expressed in all four libraries (i.e. the first 15 contigs in Table 2). RT-PCR was conducted with RNA pooled from all four libraries to establish the orientation of the template and to verify that cDNA synthesis was required for amplification. Once established, RT-PCR was conducted with RNA derived from *S*. *invicta* colonies (worker ants) field-collected from around Gainesville, Florida, (*n* = 27 from 3 locations) to determine whether the sequence was present in the U.S. field population and, if so, its prevalence. This experiment also provided additional confirmation of a potential viral origin. If amplification was observed in 100% of the samples, it was assumed that the sequence was of host origin and further experiments with the sequence were terminated. Viral infections rarely exhibit an incidence of 100% among field-collected arthropods [31].

Finally, to establish whether Contig_29 (SINV-5) was actively replicating in *S*. *invicta*, strand-specific RT-PCR was conducted to detect the replicative genome strand by the modified method of Craggs et al. [32]. Pooled total RNA (50 ng) used for library creation was mixed with 10 mM dNTPs, 1 μM of tagged reverse oligonucleotide primer p1600TAG and heated to 65°C for 5 minutes. First strand buffer and Superscript reverse transcriptase (Invitrogen, Carlsbad, CA) were then added and the reaction mixture was incubated at 55°C for 30 minutes before inactivating the RT at 70°C for 15 minutes. Unincorporated cDNA oligonucleotides were digested with 10 units of Exonuclease I (New England Biolabs, Ipswich, MA) at 37°C for 1 hour. The reaction was terminated by heating to 80°C for 20 minutes. PCR was subsequently conducted with minus-strand specific cDNA as template. The reaction was conducted in a 25 μl volume containing 2 mM MgCl_2_, 200 μM dNTP mix, 0.5 units of Platinum Taq DNA polymerase (Invitrogen, Carlsbad, CA), 0.2 μM of each oligonucleotide primer p1601 and TAG (S1 Table), and 5 μl of the cDNA preparation. The temperature cycling program was 1 cycle at 94° C for 2 minutes, 35 cycles of 94°C for 15 seconds, 59°C for 15 seconds, 68°C for 40 seconds, and 1 cycle of 68°C for 5 minutes. PCR products were separated on an agarose gel (1%) and visualized by SYBR-safe (Invitrogen, Carlsbad, CA) staining. Plus strand RT-PCR was included as a positive control as well as a non-template negative control.

## Results

**Fig 2:**
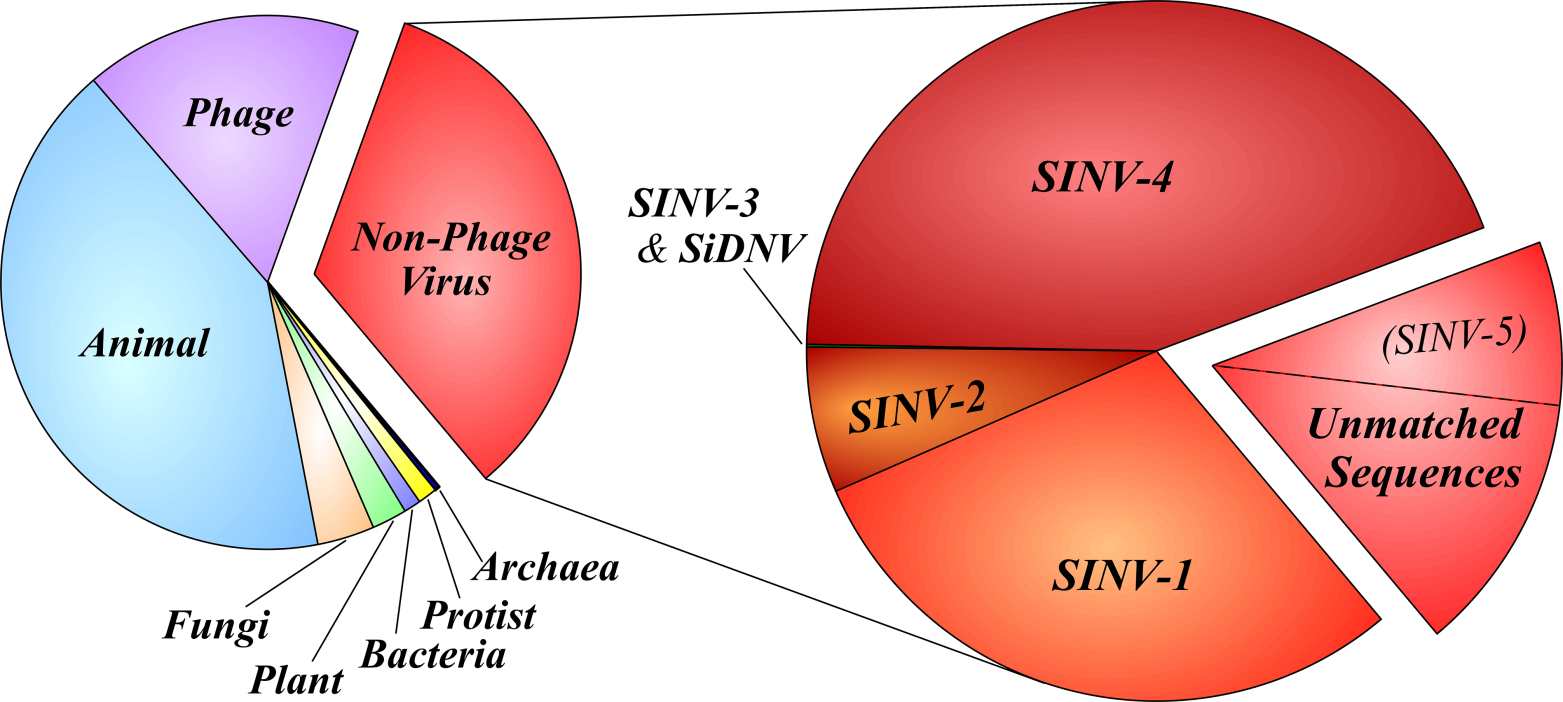
Graphical summary of broad taxonomic assignments of RNA sequences from the four combined gene libraries (Left) which did not match the *Solenopsis invicta* fire ant genome. These libraries were derived from 182 *S*. *invicta* colonies sampled from the 25 locations shown in Fig 1. Phage sequences were not of interest because they infect bacteria. The expanded pie chart (Right) shows the percentage of non-phage virus sequences matching known *S*. *invicta* viruses and those remaining sequences with significant viral identity. Note that both SINV-3 and SiDNV only accounted for a very small percentage of matched contigs. The dashed line in the unmatched section shows the proportion of sequences belonging to the new fire ant virus (SINV-5) described in this paper.

**Fig 3:**
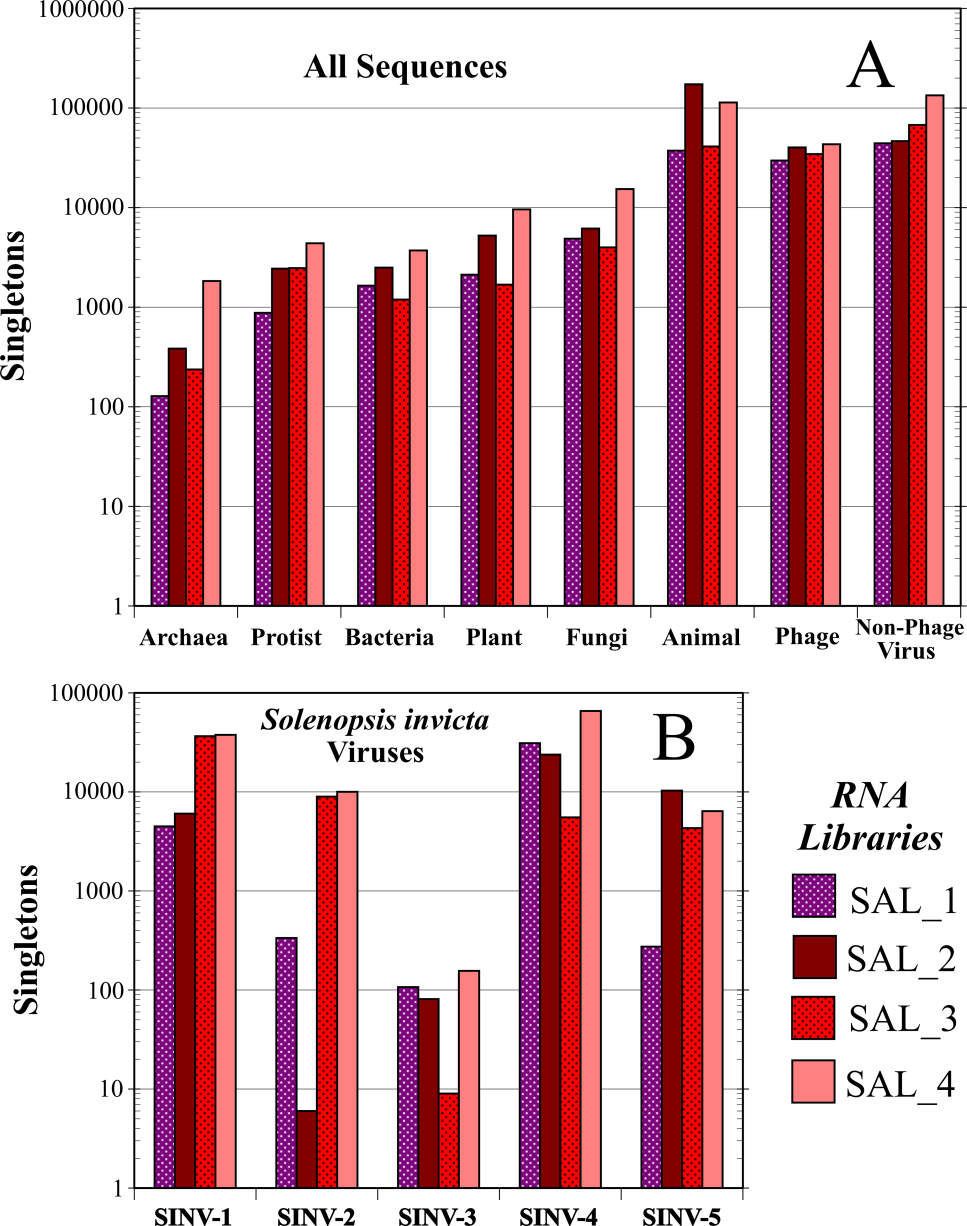
Geographic variation of singleton expression levels of sequences for each RNA library (see Fig 1 for map of collection locations and RNA library groupings). Sequences were binned according to broader taxonomic categories (Top) and known or newly described *Solenopsis invicta* viruses (Bottom). Note that expression levels are shown in log scales.

Fig 2 summarizes the annotation of the *S*. *invicta*-unmatched sequences. Phage and non-phage virus categories comprised 50.2% of the total number of non-*S*. *invicta* sequences (*n* = 876,528). The number of non-phage virus sequences was 292,499, or 33.4% of the total. Surprisingly, phage were well represented despite low numbers of bacterial sequences. However, those phage sequences detected exhibited high expression levels and were limited to the *Microviridae* (ssDNA). The remaining sequences were annotated to the following taxa: Animal (41.7%), Fungi (3.4%), Plant (2.1%), Protist (1.1%), Bacteria (1.0%), and Archaea (0.3%). The relative number of sequences across these broad taxonomic categories was fairly consistent among the four gene libraries (Fig 3A). Detection of a high percentage of animal-related sequences were largely fire ant sequences that were not filtered out during the fire ant matching phase.

Further winnowing was accomplished by removing the known *S*. *invicta* virus sequences from the non-phage virus pool (Fig 2). The total number of *S*. *invicta* virus-matched sequences was 230,555 (i.e., 78.8% of the non-phage sequences). SINV-4 (*n* = 126,086) represented the largest fraction of *S*. *invicta* known virus sequences in the pooled libraries, followed by SINV-1 (*n* = 84,769), SINV-2 (*n* = 19,338), SINV-3 (*n* = 353), and SiDNV (*n* = 9) (Fig 2). The small number of SiDNV sequences may be explained because SiDNV is a DNA virus which would normally only produce RNA while actively replicating. Conversely, many RNA viruses would be detected while replicating or not. Unlike the broader taxonomic assignments, the prevalence of *S*. *invicta* viruses varied by several orders of magnitude across each library/region (Fig 3B).

The *S*. *invicta* virus-unmatched sequences (*n* = 61,944) were assembled *de novo* yielding 171 contiguous sequences (comprised of 55,677 singletons) with significant identity to non-plant virus genes (Table 2 and S2 Table). An additional 30 contiguous sequences were assembled from 633 singletons, which showed significant identity to non-virus genes and 35 contiguous sequences (596 singletons) with significant plant virus identity, all of which were excluded from further examination. The remaining singletons and contiguous sequences (*n* = 5,038) were also excluded from further analysis because they either did not match any sequence in the GenBank database and/or were less than 100 nucleotides in length.

Among the 171 contiguous sequences with significant identity to non-plant virus genes, Table 2 summarizes those considered most likely to be of viral origin (*n* = 38) and using *S*. *invicta* as host. This assumption was based on the total sequence expression representation, representation across the libraries (i.e., by geography), and contig size. Expression representation has been used successfully to detect pathogens and is simply based on the fact that actively replicating genes (e.g., from viruses infecting a host) will be highly represented in non-normalized gene libraries [33]. Among the 38 sequences in Table 2 exhibiting significant identity with viral sequences from the GenBank database, fifteen (9%) were represented in all four gene libraries and were composed of 46,845 of the 55,677 singletons. Thus, these fifteen contiguous sequences alone accounted for 84% of the singletons assembled with non-plant virus identity and were considered high likelihood viral prospects. Nine contigs contained sequences detected in three of the four libraries and were composed of 1,276 singletons; eight contigs contained sequences detected in two of the four libraries and were composed of 927 singletons. Finally, six contigs contained sequences detected in a single library, but were highly represented within the single library (composed of 5,180 singletons). In total, these 38 contigs contained 54,228 singletons, or 97.4% of all sequences of those with non-plant virus identity. All of these sequences exhibited identity to viruses with RNA genomes, the majority of which to the *Dicistroviridae* (*n* = 23). Fourteen of the sequences exhibited identity with unclassified viruses and one with a virus in the *Nodaviridae* (i.e., Contig_51).

**Fig 4:**
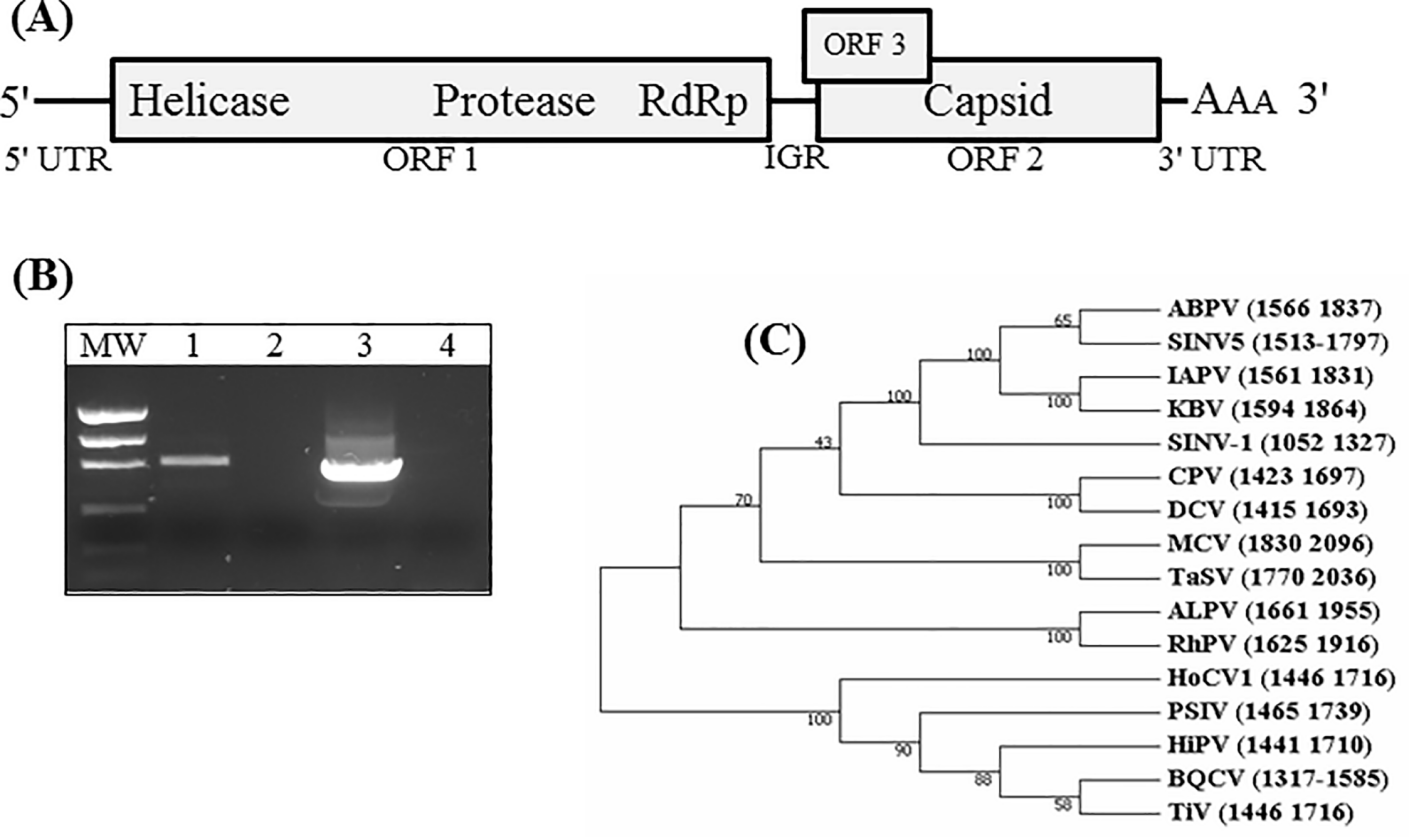
**SINV-5 genome architecture (A), detection of replicative genome strand (B), and phylogenetic analysis of the RNA-dependent RNA polyermase of SINV-5 and known dicistroviruses (C).** (A) The SINV-5 genome is represented by the center black line and open reading frames by rectangles. Proteins with identity to an RNA helicase, protease, and RNA-dependent RNA polymerase (non-structural proteins) were detected within ORF 1 and those with identity to virus capsid proteins were detected in ORF 2. A short, overlapping ORF3 at the 5' end of ORF2 provides support for Aparavirus placement. (B) RT-PCR amplification of each SINV-5 genome strand. Replicative strand (lane 1), corresponding control (lane 2), positive strand (lane 3) and corresponding control (lane 4). (C) The evolutionary relationship of SINV-5 with known dicistroviruses by comparison of the RNA-dependent RNA polymerase from each virus inferred using the Neighbor-Joining method [43]. The percentage of replicate trees in which the associated taxa clustered together in the bootstrap test (500 replicates) are shown next to the branches [44]. Only the most conserved region of the RdRp was aligned and a total of 243 positions were included in the final dataset (exact positions for the translated ORF1 are indicated in the phylogenetic tree within parentheses by each taxa). Evolutionary analyses were conducted in MEGA7 [45]. Abbreviations and GenBank Accession numbers for regions of the translated ORF1 (shown in graph) used for analysis include SINV-5 (Solenopsis invicta virus 5; MF593921), ABPV (Acute bee paralysis virus; NC002548), IAPV (Israeli acute bee paralysis virus; NC009025), KBV (Kashmir bee virus; NC004807), SINV-1 (Solenopsis invicta virus 1; NC006559), CPV (Cricket paralysis virus; NC003924), DCV (Drosophila C virus; NC001834), MCV (Mud crab virus; NC014793), TaSV (Taura syndrome virus; NC003005), ALPV (Aphid lethal paralysis virus; NC004365), RhPV (Rhopalosiphum padi virus; NC001874), HoCV (Homalodisca coagulata virus 1; NC008029), PSIV (Plautia stali intestine virus; NC003779), HiPV (Himetobi P virus; NC003782), BQCV (Black queen cell virus; NC003784), TrV (Triatoma virus; NC003783).

Sequences with the highest level of expression and represented in all four gene libraries (*n* = 15; Table 2) were considered the most likely prospects to be of viral origin (henceforth referred as *high likelihood viral prospects*). Therefore, we focused our effort on these fifteen contigs (Table 2). Contig_29 exhibited the highest expression level overall and across all four gene libraries; this sequence appeared to be a near complete virus genome. The large contig sequence was 9,030 nucleotides in length and BLASTX analysis [28] indicated that the sequence had significant identity to Israeli acute paralysis virus (IAPV) and other RNA viruses in the *Dicistroviridae*. Sanger re-sequencing and RACE reactions revealed a 9,313 nucleotide polyadenylated genome containing two, in-frame, open reading frames (ORFs) separated and flanked by untranslated regions, and a short, overlapping ORF at the 5' end of ORF 2 (Fig 4A). The 5'-proximal ORF contained domains with identity to RNA helicase (pfam00910), virus peptidase (pfam12381), and RNA-dependent RNA polymerase (pfam00680). The 3'-proximal ORF contained domains with identity to CRPV capid proteins (pfam08762) and the capsid protein, VP4, from dicistroviruses (pfam 11492). These characteristics indicate that this genome sequence represents a new dicistrovirus [34]. The virus is provisionally named, Solenopsis invicta virus 5 (SINV-5) and the sequence deposited in GenBank under accession number MF593921. Further evaluation of the SINV-5 genome revealed that the replicative strand was detected in *S*. *invicta* (from Argentinean colonies). The presence of a replicative genome strand and a high expression level of SINV-5 sequences detected in all four gene libraries indicate that *S*. *invicta* likely serves as host for the virus (Fig 4B). Phylogenetic analysis of the conserved RdRp region of ORF 1 of SINV-5 with the known *Dicistroviridae* species shows that SINV-5 assorts with dicistroviruses within the Aparavirus genus, near SINV-1 (Fig 4C). The short, overlapping ORF at the 5' end of the structural ORF (ORF 2) provides further support for the Aparavirus placement.

Contiguous sequences (specifically, Contig_66, Contig_30 and Contig_16) also exhibited high expression levels and were represented in all four libraries. These contigs also exhibited significant identity to dicistrovirus non-structural and structural proteins and likely represent new virus species. The remaining 11 contigs, Contig_70 to Contig_80 (Table 2), had lower overall expression levels, but were considered high likelihood virus prospects because they were detected in all four libraries (geographic regions) and exhibited significant identity with viral genes.

Among the fifteen high likelihood viral sequences identified (Table 2), PCR amplification only occurred after reverse transcription (Table 3). No amplification was detected without reverse transcription confirming that these templates were RNA. A small number of field-collected *S*. *invicta* colonies from Florida (*n* = 27) were also examined by RT-PCR to determine whether any of the fifteen high likelihood viral sequences were present in the U.S. *S*. *invicta* population (Table 3). Five of the templates were apparently present in the U.S. population ranging in prevalence from 15 to 56%. However, the majority were not detected in U.S. *S*. *invicta* samples.

**Table 3:** gs composed of sequences from all four gene libraries evaluated by RT-PCR and PCR. Conti Reverse transcription was required for successful amplification of all contigs. RT-PCR was also conducted with RNA obtained from *S*. *invicta* colonies (*n* = 27) collected in the U.S. to determine if the contig was present in the introduced range.

Contig designation	PCR amplification with cDNA synthesis	PCR amplification without cDNA synthesis	Presence in U.S. (%)
Contig_29 *(SINV-5)*	Yes	No	Not detected
Contig_66	Yes	No	56
Contig_30	Yes	No	56
Contig_16	Yes	No	56
Contig_70	Yes	No	26
Contig_58	Yes	No	Not detected
Contig_13	Yes	No	15
Contig_21	Yes	No	Not detected
Contig_83	Yes	No	Not detected
Contig_55	Yes	No	Not detected
Contig_82	Yes	No	Not detected
Contig_15	Yes	No	Not detected
Contig_27	Yes	No	Not detected
Contig_17	Yes	No	Not detected
Contig_80	Yes	No	Not detected

## Discussion

In an effort to discover new viral pathogens to possibly control *S*. *invicta* in the U.S., we collected samples from 182 nests from four distinct geographic areas across the Formosa region of Argentina, created gene libraries from each of these pooled groups, and sequenced each of them by the Illumina Miseq method. Through a series of winnowing methods, 171 contiguous sequences with significant viral identity were ultimately identified as viral candidates.

Among these 171 possibilities, we focused on 15 contigs because they exhibited the highest expression levels and were detected in all 4 geographic regions (Table 2). They were analyzed in an attempt to establish their origin—whether viral, host, or otherwise. *Solenopsis invicta* is an omnivorous insect, so viruses infecting prey or plant food items must be identified and excluded from consideration. We largely employed the step-by-step decision tree reported previously [35] as a general guide to determine the likelihood that a given sequence was of viral origin. Based on previous studies [18, 36], this winnowing method significantly improves virus identification and discovery. In addition to this decision tree, the relative prevalence of each contig/sequence was considered supporting evidence for viral replication and host status. Because no form of mRNA subtraction was conducted before library preparation, the representation of each transcript was expected to be relatively proportional to the actual number of sequences present in the sample. Basically, ingested viruses would likely be represented by fewer sequence copies. Conversely, replicating viruses would be indicated by higher sequence copy numbers. Furthermore, sequences found in more than a single region/library would provide further support that it was, in fact, of virus origin and infecting *S*. *invicta*.

However, low representation does not preclude the possibility that a sequence is not from a virus infecting *S*. *invicta*. Indeed, low gene/genome copy number has been reported for actively replicating viruses that have detrimental effects on their hosts [22]. Additionally, some RNA viruses, which can be virulent and highly represented, may occur in low numbers because of seasonal variation, low host infection rates, or other unknown factors [37, 38].

One nearly complete virus genome was assembled among the high likelihood virus contigs (Contig_29; Table 2). RACE reactions using this contig as template and subsequent Sanger re-sequencing resulted in a complete virus genome. We have provisionally named this virus sequence SINV-5. It exhibits characteristics consistent with dicistroviruses in the Aparavirus genus, including, a monopartite genome containing two, in-frame, ORFs that are flanked and separated by untranslated regions, a short, overlapping ORF at the 5' end of ORF 2, and a polyadenylated 3' terminus. The 5'-proximal ORF contained domains with identity to RNA helicase, protease, and RNA-dependent RNA polymerase, and the 3'-proximal ORF contained domains with identity to virus capsid proteins. SINV-5 was only detected in *S*. *invicta* colonies from Argentina; it was not detected in a limited number of *S*. *invicta* colonies from three locations in the U.S. In addition to the genomic architecture, phylogenetic analysis of the RdRp further supports a taxonomic assignment of SINV-5 in the *Dicistroviridae* (Fig 4C). Taken together, high expression levels, detection of a replicative strand, and phylogeny of SINV-5 indicate that *S*. *invicta* serves as the host for this novel virus (Fig 4B). Depending on the impact of this virus on fire ants and it host specificity, it may be a candidate for introduction into the U.S. as a classical biological control agent for *S*. *invicta*.

We also discovered what will likely be a second virus among the unidentified contigs in Table 2. Two contigs (Contig_66 and Contig_30) exhibited identity with Aphid lethal paralysis virus (ALPV) structural and non-structural proteins, respectively (Table 2). Because ALPV is a dicistrovirus, we postulated that these two fragments may have been part of the same genome, but were lacking sequence linking them. The number of sequences comprising each of these contigs further supported this notion. Notice that in every library (SAL-1, -2, -3, and -4), more sequences comprised Contig_66 than Contig_30 (Table 2). This relationship would be expected in a host in which a dicistrovirus was actively replicating. Specifically, there would be a molar excess of capsid proteins compared with non-structural proteins. RT-PCR with a reverse primer (P1617) specific for Contig_30 and forward primer (P1631) specific for Contig_66 produced an amplicon (~3 kbp), whose sequence linked the two contigs. The joined contig was 4,505 nucleotides in length. This sequence was detected in both Argentinean and U.S. *S*. *invicta* populations (Table 3) and likely represents another virus as the replicative strand of this genome was also detected in U.S. and Argentinean *S*. *invicta* colonies (Data not shown).

A majority of the contigs tested by RT-PCR (10/15; Table 3) were only detected in the RNA libraries from Argentina. This fits well with the hypothesis that most of the natural enemies of *S*. *invicta* were left behind in South America when it was accidently introduced into the U.S. [7]. Nevertheless, it is of interest that 4 of the 5 *S*. *invicta* viruses discovered to date, plus the likely one just mentioned above, plus 5 of 15 likely viral contigs tested (Table 3) have been found in both North and South American populations of *S*. *invicta*. Similarly, the microsporidian pathogen *Kneallhazia solenopsae* has been found infecting fire ants on both continents, but the microsporidian, *Vairimorpha invictae* is only found in South America [39]. This frequency of pathogens found on both continents supports the conclusion that multiple colonies of *S*. *invicta* were introduced into the U.S., perhaps over a period of years [1] because it is highly unlikely that a single invading colony or only a few colonies would carry this many pathogens [37]. It also appears that most of the viruses found in the U.S. were naturally common in native South American fire ant populations (Fig 3). Certainly, at least 3 of the viruses (SINV-1, SINV-2, SINV-3) can be seasonally abundant in U.S. populations [37]. Another possible explanation for viral pathogens on both continents is that some may be generalists with a wide host range that already occurred in the U.S. on other ant species, especially some of the native fire ants. However, the two viruses tested to date (SINV-1 and SINV-3) appear to have originated from South America and are specific for *S*. *invicta* [38, 40, 41].

While high expression levels are a logical place to start to discover new viruses from next generation sequencing data, low expression levels do not necessarily preclude a sequence from consideration. Indeed, SINV-2 was shown to exhibit comparatively low expression in *S*. *invicta* queens, yet had a profound impact on fecundity and gene expression during colony founding [22]. In fact, low copy sequences from gene libraries have previously resulted in virus discovery from the tawny crazy ant, *Nylanderia fulva* [35]. S2 Table contains 133 contiguous sequences with significant viral identity, all comprised of fewer than 50 singletons. Most of these sequences exhibited significant identity with RNA viral genomes, however, seven sequences showed identity to DNA viral genomes (5 single stranded and 2 double stranded). Thus, there are many possible virus leads resulting from this study that require additional investigation to establish their origin and relationship to *S*. *invicta*. All sequences have been deposited in Genbank to facilitate and encourage the discovery of additional viral pathogens of *S*. *invicta* in South America. Hopefully, new homologies can be discovered by employing new search algorithms and by periodically reanalyzing these libraries as new viral sequences are added to sequence databases.

In conclusion, our ongoing metagenomics/next generation sequencing efforts [14] have been very successful. In this study, we were able to match 79% of the non-phage virus contigs to known fire ant viruses (Fig 2). We also expanded the virome of *S*. *invicta* by discovery of a new *S*. *invicta*-infecting virus, provisionally named, SINV-5. SINV-5 is of particular interest because it does not appear to occur in the introduced U.S. range of *S*. *invicta* and therefore may be able to be released as a self-sustaining classical biological control agent for these invasive ants in the U.S. Another apparent virus sequence (Contigs_30 and 66; Table 2) appears to be a new virus of *S*. *invicta*, although the genome is incomplete. To date, we have discovered and sequenced the entire genomes of 6 viruses in South American fire ants (i.e., SINV-1, -2, -3, -4, -5 and SiDNV). By way of comparison, 31 viruses have been characterized from honey bees [42]. Future work with SINV-5 and other newly discovered fire ant viruses will focus on their pathogenicity, host specificity, and seasonality in order to assess their potential for use as self-sustaining biocontrol agents and/or biopesticides.

## Supporting information

S1 Table Oligonucleotide primers and their purpose from experiments in this study. (DOCX)

S2 Table Contiguous sequences comprised of fewer than 50 singletons with significant viral identity by BLASTX analysis of the GenBank database from RNA libraries created from *Solenopsis invicta* worker ants. Contigs were first sorted in descending order based on the number of the sequences comprising it, followed by the libraries represented.
(DOCX)

## References

[1] Tschinkel WR. The fire ants. Cambridge: The Belknap Press of Harvard University Press; 2006. 723 p.

[2] Caldera EJ, Ross KG, DeHeer CJ, Shoemaker D. Putative native source of the invasive fire ant *Solenopsis invicta* in the USA. Biological Invasions. 2008;10(8):1457–79.

[3] LeBrun EG, Tillberg CV, Suarez AV, Folgarait PJ, Smith CR, Holway DA. An experimental study of competition between fire ants and Argentine ants in their native range. Ecology. 2007;88(1):63–75. doi: 10.1890/0012-9658(2007)88[63:Aesocb]2.0.Co;2 PubMed PMID: WOS:000245668300010. 17489455 10.1890/0012-9658(2007)88[63:aesocb]2.0.co;2

[4] Calcaterra LA, Livore JP, Delgado A, Briano JA. Ecological dominance of the red imported fire ant, *Solenopsis invicta*, in its native range. Oecologia. 2008;156(2):411–21. Epub 2008/02/29. doi: 10.1007/s00442-008-0997-y . 18305962 10.1007/s00442-008-0997-y

[5] Pereira RM. Areawide suppression of fire ant populations in pastures: project update. J Agric Urban Entomol. 2003;20(2):123–30.

[6] Porter SD, Fowler HG, Mackay WP. Fire ant mound densities in the United States and Brazil (Hymenoptera: Formicidae). Journal of Economic Entomology. 1992;85(4):1154–61.

[7] Porter SD, Williams DF, Patterson RS, Fowler HG. Intercontinental differences in the abundance of *Solenopsis* fire ants (Hymenoptera: Formicidae): escape from natural enemies? Environmental Entomology. 1997;26(2):373–84.

[8] Yang CC, Yu YC, Valles SM, Oi DH, Chen YC, Shoemaker D, et al Loss of microbial (pathogen) infections associated with recent invasions of the red imported fire ant *Solenopsis invicta*. Biological Invasions. 2010;12(9):3307–18. doi: 10.1007/s10530-010-9724-9 PubMed PMID: ISI:000280892600041.

[9] Callcott AM, Collins HL. Invasion and range expansion of imported fire ants (Hymenoptera: Formicidae) in North America from 1918–1995. Florida Entomologist. 1996;79(2):240–51.

[10] Schmid-Hempel P. Parasites in Social Insects. Princeton: Princeton University Press; 1998. 409 p.

[11] Oi DH, Valles SM. Fire ant control with entomopathogens in the USA In: Hajek AE, Glare TR, O'Callaghan M, editors. Use of microbes for control and eradication of invasive arthropods. New York: Springer Science; 2009 p. 237–58.

[12] Callcott AM, Porter SD, Weeks RD Jr., Graham LC, Johnson SJ, Gilbert LE. Fire ant decapitating fly cooperative release programs (1994–2008): Two *Pseudacteon* species (*P*. *tricuspis*, *P*. *curvatus*) rapidly expand across imported fire ant populations in the southeastern United States. Journal of Insect Science. 2010;11(19):1–25. 10.1673/031.011.0119 PMC3281391 21526930

[13] Porter SD, Kumar V, Calcaterra LA, Briano JA, Seal DR. Release and establishment of the little decapitating fly *Pseudacteon cultellatus* (Diptera: Phoridae) on imported fire ants (Hymenoptera: Formicidae) in Florida. Florida Entomologist. 2013;96(4):1567–73. PubMed PMID: WOS:000329082500040.

[14] Valles SM. Positive-strand RNA viruses infecting the red imported fire ant, *Solenopsis invicta*. Psyche. 2012;2012:1–14. doi: 10.1155/2012/821591

[15] Briano J, Calcaterra L, Varone L. Fire ants (*Solenopsis* spp.) and their natural enemies in southern South America. Psyche. 2012;2012:Article ID 198084, 1–19.

[16] Lacey LA, Frutos R, Kaya HK, Vail P. Insect pathogens as biological control agents: Do they have a future? Biological Control. 2001;21(3):230–48.

[17] Chen Y, Becnel JJ, Valles SM. RNA viruses infecting pest insects In: Vega F, Kaya HK, editors. Insect Pathology. 2nd ed. Amsterdam: Elsevier; 2012 p. 133–70.

[18] Valles SM, Strong CA, Dang PM, Hunter WB, Pereira RM, Oi DH, et al A picorna-like virus from the red imported fire ant, *Solenopsis invicta*: initial discovery, genome sequence, and characterization. Virology. 2004;328(1):151–7. Epub 2004/09/24. doi: 10.1016/j.virol.2004.07.016 S0042-6822(04)00491-X [pii]. . 15380366 10.1016/j.virol.2004.07.016

[19] Valles SM, Strong CA, Hashimoto Y. A new positive-strand RNA virus with unique genome characteristics from the red imported fire ant, *Solenopsis invicta*. Virology. 2007;365(2):457–63. Epub 2007/05/05. doi: S0042-6822(07)00212-7 [pii] doi: 10.1016/j.virol.2007.03.043 . 17477949 10.1016/j.virol.2007.03.043

[20] Valles SM, Hashimoto Y. Isolation and characterization of *Solenopsis invicta* virus 3, a new postive-strand RNA virus infecting the red imported fire ant, *Solenopsis invicta*. Virology. 2009;388(2):354–61. doi: 10.1016/j.virol.2009.03.028 19403154 10.1016/j.virol.2009.03.028

[21] Olendraite I, Lukhovitskaya NI, Porter SD, Valles SM, Firth AE. Polycipiviridae: a proposed new family of polycistronic picorna-like RNA viruses. J Gen Virol. In press. 10.1099/jgv.0.000902 PMC5656759 28857036

[22] Manfredini F, Shoemaker D, Grozinger CM. Dynamic changes in host-virus interactions associated with colony founding and social environment in fire ant queens (*Solenopsis invicta*). Ecol Evol. 2016;6(1):233–44. doi: 10.1002/ece3.1843 . 26811788 10.1002/ece3.1843 PMC4716520

[23] Valles SM, Porter SD, Choi MY, Oi DH. Successful transmission of Solenopsis invicta virus 3 to *Solenopsis invicta* fire ant colonies in oil, sugar, and cricket bait formulations. Journal of Invertebrate Pathology. 2013;113(3):198–204. doi: 10.1016/j.jip.2013.04.003 PubMed PMID: WOS:000320633900002. 23602901 10.1016/j.jip.2013.04.003

[24] Valles SM, Porter SD, Firth AE. Solenopsis invicta virus 3: pathogensis and stage specificity in red imported fire ants. Virology. 2014;461:66–71. 10.1016/j.virol.2014.04.026 25010271

[25] Valles SM, Shoemaker D, Wurm Y, Strong CA, Varone L, Becnel JJ, et al Discovery and molecular characterization of an ambisense densovirus from South American populations of *Solenopsis invicta*. Biological Control. 2013;67(3):431–9. doi: 10.1016/j.biocontrol.2013.09.015 PubMed PMID: ISI:000327720100016.

[26] Gandon S, Van Zandt PA. Local adaptation and host–parasite interactions. Trends in Ecology & Evolution. 1998;13(6):214–6. http://dx.doi.org/10.1016/S0169-5347(98)01358-5. 21238271 10.1016/s0169-5347(98)01358-5

[27] Li H, Durbin R. Fast and accurate short read alignment with Burrows-Wheeler transform. Bioinformatics. 2009;25(14):1754–60. doi: 10.1093/bioinformatics/btp324 . 19451168 10.1093/bioinformatics/btp324 PMC2705234

[28] Altschul SF, Madden TL, Schaffer AA, Zhang J, Zhang Z, Miller W, et al Gapped BLAST and PSI-BLAST: a new generation of protein database search programs. Nucleic Acids Research. 1997;25(17):3389–402. Epub 1997/09/01. doi: gka562 [pii]. . 9254694 10.1093/nar/25.17.3389 PMC146917

[29] Mukherjee S, Huntemann M, Ivanova N, Kyrpides NC, Pati A. Large-scale contamination of microbial isolate genomes by Illumina PhiX control. Stand Genomic Sci. 2015;10:18 doi: 10.1186/1944-3277-10-18 . 26203331 10.1186/1944-3277-10-18 PMC4511556

[30] Huang X, Madan A. CAP3: A DNA Sequence Assembly Program. Genome Res. 1999;9(9):868–77. doi: 10.1101/gr.9.9.868 10508846 10.1101/gr.9.9.868 PMC310812

[31] Fuxa JR, Tanada Y. Epizootiology of insect diseases. New York: John Wiley and Sons; 1987. 555 p.

[32] Craggs JK, Ball JK, Thomson BJ, Irving WL, Grabowska AM. Development of a strand-specific RT-PCR based assay to detect the replicative form of Hepatitis C virus RNA. Journal of Virological Methods. 2001;94(1–2):111–20. Epub 2001/05/05. doi: S0166093401002816 [pii]. . 11337045 10.1016/s0166-0934(01)00281-6

[33] Johansson H, Dhaygude K, Lindstrom S, Helantera H, Sundstrom L, Trontti K. A metatranscriptomic approach to the identification of microbiota associated with the ant *Formica exsecta*. PLoS One. 2013;8(11):e79777 doi: 10.1371/journal.pone.0079777 . 24260298 10.1371/journal.pone.0079777 PMC3832538

[34] Valles SM, Chen Y, Firth AE, Guerin DM, Hashimoto Y, Herrero S, et al ICTV Virus Taxonomy Profile: Dicistroviridae. J Gen Virol. 2017;98(3):355–6. doi: 10.1099/jgv.0.000756 . 28366189 10.1099/jgv.0.000756 PMC5797946

[35] Valles SM, Oi DH, Yu F, Tan XX, Buss EA. Metatranscriptomics and pyrosequencing facilitate discovery of potential viral natural enemies of the invasive Caribbean crazy ant, *Nylanderia pubens*. PLoS ONE. 2012;7(2):e31828 Epub 2012/03/03. doi: 10.1371/journal.pone.0031828 PONE-D-11-23360 [pii]. . 22384082 10.1371/journal.pone.0031828 PMC3288052

[36] Valles SM, Strong CA, Hunter WB, Dang PM, Pereira RM, Oi DH, et al Expressed sequence tags from the red imported fire ant, *Solenopsis invicta*: annotation and utilization for discovery of viruses. Journal of Invertebrate Pathology. 2008;99(1):74–81. Epub 2008/03/11. doi: S0022-2011(08)00018-9 [pii] doi: 10.1016/j.jip.2008.01.004 . 18329665 10.1016/j.jip.2008.01.004

[37] Valles SM, Oi DH, Porter SD. Seasonal variation and the co-occurrence of four pathogens and a group of parasites among monogyne and polygyne fire ant colonies. Biological Control. 2010;54(3):342–8.

[38] Valles SM, Strong CA, Oi DH, Porter SD, Pereira RM, Vander Meer RK, et al Phenology, distribution, and host specificity of *Solenopsis invicta* virus-1. Journal of Invertebrate Pathology. 2007;96(1):18–27. Epub 2007/04/07. doi: S0022-2011(07)00038-9 [pii] doi: 10.1016/j.jip.2007.02.006 . 17412359 10.1016/j.jip.2007.02.006

[39] Oi DH, Valles SM, Porter SD. The fire ant pathogen *Vairimorpha invictae* (Microsporidia: Burenellidae) not detected in Florida. Florida Entomologist. 2012;95(2):506–8.

[40] Porter SD, Valles SM, Oi DH. Host specificity and colony impacts of Solenopsis invicta virus 3. Journal of Invertebrate Pathology. 2013;114:1–6. doi: 10.1016/j.jip.2013.04.013 23665158 10.1016/j.jip.2013.04.013

[41] Porter SD, Valles SM, Wild AL, Dieckmann R, Plowes NJR. *Solenopsis invicta virus 3*: further host-specificity tests with native *Solenopsis* ants (Hymenoptera: Formicidae). Florida Entomologist. 2015;98:122–5.

[42] Remnant EJ, Shi M, Buchmann G, Blacquirere T, Holmes EC, Beekman M, et al A diverse range of novel RNA viruses in geographically distinct honey bee populations. Journal of Virology. 2017;(In press). 10.1128/JVI.00158-17 PMC5533899 28515299

[43] Saitou N, Nei M. The neighbor-joining method: a new method for reconstructing phylogenetic trees. Molecular Biology and Evolution. 1987;4(4):406–25. Epub 1987/07/01. doi: 10.1093/oxfordjournals.molbev.a040454 . 3447015 10.1093/oxfordjournals.molbev.a040454

[44] Felsenstein J. Confidence Limits on Phylogenies: An Approach Using the Bootstrap. Evolution. 1985;39(4):783–91. doi: 10.1111/j.1558-5646.1985.tb00420.x . 28561359 10.1111/j.1558-5646.1985.tb00420.x

[45] Kumar S, Stecher G, Tamura K. MEGA7: Molecular Evolutionary Genetics Analysis Version 7.0 for Bigger Datasets. Mol Biol Evol. 2016;33(7):1870–4. doi: 10.1093/molbev/msw054 . 27004904 10.1093/molbev/msw054 PMC8210823

