# Supplementary material for: Prospecting for viral natural enemies of the fire ant Solenopsis invicta in Argentina
Source: PLoS One. 2018 Feb 21;13(2):e0192377. doi: 10.1371/journal.pone.0192377 (PMC5821328; doi:10.1371/journal.pone.0192377)
Supplement: S1 Table — (DOCX) [file pone.0192377.s001.docx]

**Supplementary Table 1.** Oligonucleotide primers and their purpose from experiments in this study.

| **Designation** | **Purpose/use** | **Sequence** |
| --- | --- | --- |
| P1600 | SINV-5 re-sequencing/US detection/orientation | TAGATGAAGTCTAGTAGTCTCCAAATATTCCCGTGA |
| p1600TAG | Replicative strand detection | GGCCGTCATGGTGGCGAATAATAGATGAAGTCTAGTAGTCTCCAAATATTCCCGTGA |
| P1601 | 5'RACE/SINV-5 re-sequencing/orientation | AGATACTTTTCCCAAATACTTCTTAATCATACGGAAT |
| P1604 | 3'RACE | ATTTGGAACGAAACAGAGATGGACCTATTCCAT |
| P1608 | US detection/orientation | GAACGAGGTGCCCTATTTAGGGTGTGGA |
| P1610 | US detection/orientation | ACTCCAGGATAGGCTGTAACACCTGCTCTT |
| P1614 | US detection/orientation | TTGGTTTGATAACATCACGTTAGTAGGACCAACA |
| P1615 | US detection/orientation | TAGTCAATCTCCATCTCATCCACTCCTGAC |
| P1616 | US detection/orientation | AGTCACCAGCCAATGCTCTAATGTGATC |
| P1617 | Sequence verification/US detection/orientation | ACGACACAGATACCATCGACTGTTCAGTATCACT |
| P1625 | US detection/orientation | GTATTCAACAACTACATATCACCAGAGGTGGTAT |
| P1626 | US detection/orientation | TTCACTGGTTCATACAGCATCTGGAGTGGAC |
| P1627 | US detection/orientation | CACGTAATCAGCATTATCACGGCGATAAATA |
| P1628 | US detection/orientation | TGCTTGTCCTATTGCAGTTAAAGCACTGTC |
| P1629 | US detection/orientation | GCTATCTACAGATGTCGTTGACAATGAATTTGCA |
| P1630 | US detection/orientation | ACACACGTATGCTAGATCCATCTCATCAACTGATGA |
| P1631 | Sequence verification | TGCTATGAGTTGGATGTACACCGGAGTGACCTT |
| p1631TAG | Replicative strand detection | GGCCGTCATGGTGGCGAATAATGCTATGAGTTGGATGTACACCGGAGTGACCTT |
| P1632 | Replicative strand detection | TGAATTTAATCCTTCGACTCCTTCTACTCCTACAAT |
| P1633 | US detection/orientation | CTCGTGTCAAGGCACAAGTTGGTATTGATATTAAT |
| P1634 | US detection/orientation | ATATAATTCGTCGCGGTATCGGTAATAGGGTCTAT |
| P1635 | US detection/orientation | GAGATCCTGCATTTGACAGAGTCCAAGAGCAA |
| P1636 | US detection/orientation | TCAGCCATAATCTTGTCGTTGATTTCTATC |
| P1637 | US detection/orientation | TGACTAATGACACTGAAATTACTATCAGAGTTCCTT |
| P1638 | US detection/orientation | ATAGCATGTTTGGGATTGTTTTAGTGCTGTTGT |
| P1639 | US detection/orientation | CCAGAGTCTACCATGTGAAACCAAACTGCGAG |
| P1640 | US detection/orientation | CACTATCTGTGTCAAGCCAGGCTGATTCAGGT |
| P1641 | US detection/orientation | TGTTCGTCTCGAGAGCGTCACAATTGAT |
| P1642 | US detection/orientation | GCTCAGAGTCATTGAGCAATAGTATCCTAGCTC |
| P1643 | US detection/orientation | TCATTCTATGTCTGGTCATTTCCGTGATTTAGCTT |
| P1644 | US detection/orientation | ATATACATTGTTTTCTGTCCAGCGTATCCGTCA |
| P1645 | US detection/orientation | CAATTAGGATTAAGACCATCTGATGAAATCGAACA |
| P1646 | US detection/orientation | CGAAAAGCATCTGGGAATGTGAGTGAACT |
| P1647 | US detection/orientation | CATATCATGGAGAACAGAATTGACAACGAGCAA |
| P1648 | US detection/orientation | TACCATCTGTTTCCTCGTTTAATAAAGTTGCGCTT |
| P29_1 | SINV-5 re-sequencing | AGCAGAGATGCAGATATTTGATATGTTTGCT |
| P29_2 | SINV-5 re-sequencing | TCTTCATATTCAATGTCTTCAGCATATTTATCT |
| P29_3 | SINV-5 re-sequencing | ACGTAGAGTAGATCTATGTGCTGAGGTCAGGA |
| P29_4 | SINV-5 re-sequencing | AATGCCTCTACGATCTTGGGTTTTTGAACAGC |
| P29_5 | SINV-5 re-sequencing | AAGCTACAACTTCAGGAGATTGTGTGACACGTA |
| P29_6 | SINV-5 re-sequencing | ACACACGTGTCTTCAATTGGTTTACTTTCTCA |
| P29_7 | SINV-5 re-sequencing | AGAATTGAAGCAGCAAAGAAAGGTATTCGACTCC |
| P29_8 | SINV-5 re-sequencing | TCGTCGAAGTTGTTTGTCTCTGTCATTTGTAGT |
| P29_9 | SINV-5 re-sequencing | GGATGATACGCACAGTATTATTCAGTTTCTACAAC |
| P29_10 | SINV-5 re-sequencing | AACTGAAGTAATGGATCCCGTACCTTGTGAATACGT |
| P29_11 | SINV-5 re-sequencing | TGTCCTATAAATCATGTCAATGCGCTGATAGAA |
| P29_12 | SINV-5 re-sequencing | TGTACGTAGGGAACAACCCTACGTATAATG |
| TAG | Replicative strand detection | GGCCGTCATGGTGGCGAATAA |
