# Supplementary material for: Prospecting for viral natural enemies of the fire ant Solenopsis invicta in Argentina
Source: PLoS One. 2018 Feb 21;13(2):e0192377. doi: 10.1371/journal.pone.0192377 (PMC5821328; doi:10.1371/journal.pone.0192377)
Supplement: S2 Table — Contigs were first sorted in descending order based on the number of the sequences comprising it, followed by the libraries represented. (DOCX) [file pone.0192377.s002.docx]

**Supplementary Table 2.** Contiguous sequences comprised of fewer than 50 singletons with significant viral identity by BLASTX analysis of the GenBank database from RNA libraries created from *Solenopsis invicta* worker ants. Contigs were first sorted in descending order based on the number of the sequences comprising it, followed by the libraries represented.

| **Designation** | **Sequences comprising contig** | **Size (nt)** | **e-score** | **Query coverage (%)** | **Query identity (%)** | **Sequence identity with** | **Number of singletons in** | | | | **Genome** | **Virus family** |
| --- | --- | --- | --- | --- | --- | --- | --- | --- | --- | --- | --- | --- |
|  |  |  |  |  |  |  | **SAL_1** | **SAL_2** | **SAL_3** | **SAL_4** |  |  |
| SAL_1234_Contig_10 | 49 | 456 | 2E^-30^ | 68 | 51 | SINV-2 | 10 | 0 | 0 | 39 | ssRNA | Unclassified |
| SAL_1234_Contig_14 | 43 | 454 | 3E^-44^ | 98 | 56 | SINV-2 | 0 | 37 | 0 | 6 | ssRNA | Unclassified |
| SAL_1234_Contig_33 | 38 | 865 | 4E^-70^ | 93 | 49 | Wuhan arthropod virus 2 | 0 | 0 | 37 | 1 | RNA | Unclassified |
| SAL_1234_Contig_86 | 28 | 361 | 5E^-75^ | 99 | 98 | Aphid lethal paralysis virus | 10 | 18 | 0 | 0 | ssRNA | Dicistroviridae |
| SAL_1234_Contig_59 | 28 | 722 | 4E^-80^ | 99 | 55 | SINV-2 | 0 | 24 | 0 | 4 | ssRNA | Unclassified |
| SAL_1234_Contig_37 | 27 | 415 | 9E^-18^ | 41 | 70 | Israeli acute paralysis virus | 0 | 26 | 1 | 0 | ssRNA | Dicistroviridae |
| SAL_1234_Contig_32 | 26 | 888 | 6E^-66^ | 98 | 42 | Aphid lethal paralysis virus | 7 | 0 | 19 | 0 | ssRNA | Dicistroviridae |
| SAL_1234_Contig_62 | 23 | 392 | 4E^-31^ | 65 | 69 | Shuangao insect virus 11 | 0 | 20 | 0 | 3 | RNA | Unclassified |
| SAL_1234_Contig_41 | 23 | 797 | 4E^-42^ | 92 | 41 | Aphid lethal paralysis virus | 8 | 0 | 15 | 0 | ssRNA | Dicistroviridae |
| SAL_1234_Contig_81 | 22 | 638 | 2E^-38^ | 85 | 54 | Kashmir bee virus | 17 | 0 | 5 | 0 | ssRNA | Dicistroviridae |
| SAL_1234_Contig_24 | 18 | 531 | 1E^-107^ | 100 | 92 | Acute bee paralysis virus | 0 | 0 | 6 | 12 | ssRNA | Dicistroviridae |
| SAL_1234_Contig_54 | 17 | 629 | 2E^-57^ | 99 | 50 | Israeli acute paralysis virus | 0 | 16 | 0 | 1 | ssRNA | Dicistroviridae |
| SAL_1234_Contig_26 | 16 | 458 | 2E^-98^ | 99 | 99 | Rhopalosiphum padi virus | 0 | 0 | 3 | 13 | ssRNA | Dicistroviridae |
| SAL_1234_Contig_69 | 15 | 400 | 1E^-11^ | 75 | 38 | Hubei picorna-like virus 48 | 0 | 2 | 0 | 13 | RNA | Unclassified |
| SAL_1234_Contig_76 | 14 | 190 | 1E^-28^ | 99 | 76 | Hubei picorna-like virus 41 | 5 | 0 | 9 | 0 | RNA | Unclassified |
| SAL_1234_Contig_34 | 14 | 289 | 1E^-43^ | 97 | 78 | Drosophila C virus | 4 | 0 | 10 | 0 | ssRNA | Dicistroviridae |
| SAL_1234_Contig_23 | 12 | 161 | 9E^-13^ | 93 | 60 | Hubei orthoptera virus 1 | 0 | 0 | 6 | 6 | RNA | Unclassified |
| SAL_1234_Contig_73 | 12 | 395 | 2E^-45^ | 99 | 59 | Beihai picorna-like virus 77 | 0 | 2 | 0 | 10 | RNA | Unclassified |
| SAL_1234_Contig_5 | 9 | 289 | 1E^-27^ | 99 | 54 | Spodoptera exigua virus AKJ-2014 | 1 | 0 | 0 | 8 | ssRNA | Picornaviridae |
| SAL_1234_Contig_87 | 9 | 299 | 2E^-39^ | 88 | 76 | Hubei orthoptera virus 1 | 7 | 2 | 0 | 0 | RNA | Unclassified |
| SAL_1234_Contig_35 | 8 | 307 | 2E^-35^ | 98 | 51 | Beihai picorna-like virus 76 | 4 | 0 | 4 | 0 | RNA | Unclassified |
| SAL_1234_Contig_20 | 7 | 290 | 1E^-36^ | 99 | 68 | Hubei orthoptera virus 1 | 0 | 1 | 0 | 6 | RNA | Unclassified |
| SAL_1234_Contig_79 | 6 | 475 | 2E^-99^ | 99 | 97 | Spodoptera exigua iflavirus | 3 | 0 | 3 | 0 | ssRNA | Iflaviridae |
| SAL_1234_Contig_4 | 5 | 482 | 2E^-35^ | 95 | 45 | Spodoptera exigua virus AKJ-2014 | 0 | 2 | 0 | 3 | ssRNA | Picornaviridae |
| SAL_1234_Contig_42 | 4 | 253 | 4E^-51^ | 99 | 98 | Rhopalosiphum padi virus | 2 | 0 | 2 | 0 | ssRNA | Dicistroviridae |
| SAL_1234_Contig_6 | 4 | 254 | 7E^-38^ | 96 | 84 | Drosophila C virus | 0 | 2 | 0 | 2 | ssRNA | Dicistroviridae |
| SAL_1234_Contig_72 | 4 | 281 | 7E^-33^ | 99 | 62 | Hubei orthoptera virus 1 | 2 | 2 | 0 | 0 | RNA | Unclassified |
| SAL_1234_Contig_38 | 3 | 300 | 9E^-90^ | 84 | 54 | Hubei orthoptera virus 1 | 2 | 0 | 1 | 0 | RNA | Unclassified |
| SAL_1234_Contig_71 | 3 | 349 | 5E^-67^ | 99 | 92 | Aphid lethal paralysis virus | 0 | 2 | 1 | 0 | ssRNA | Dicistroviridae |
| SAL_1234_Contig_89 | 3 | 456 | 6E^-104^ | 100 | 97 | Black queen cell virus | 1 | 2 | 0 | 0 | ssRNA | Dicistroviridae |
| SAL_1234_Contig_67 | 2 | 290 | 1E^-17^ | 51 | 76 | Kashmir bee virus | 0 | 1 | 1 | 0 | ssRNA | Dicistroviridae |
| SAL_1234_Contig_90 | 2 | 301 | 3E^-12^ | 66 | 51 | Drosophila C virus | 1 | 0 | 0 | 1 | ssRNA | Dicistroviridae |
| SAL_4_Contig_83_32 | 32 | 403 | 2E^-40^ | 96 | 54 | Aphis glycines virus 1 | 0 | 0 | 0 | 32 | RNA | Unclassified |
| SAL_1_Contig_78_24 | 24 | 298 | 3E^-53^ | 98 | 87 | Wuhan insect virus 11 | 24 | 0 | 0 | 0 | RNA | Unclassified |
| SAL_2_Contig_57_24 | 24 | 338 | 3E^-54^ | 98 | 79 | Hubei picorna-like virus 23 | 0 | 24 | 0 | 0 | RNA | Unclassified |
| SAL_1_Contig_36_23 | 23 | 355 | 7E^-53^ | 99 | 75 | Israeli acute paralysis virus | 23 | 0 | 0 | 0 | ssRNA | Dicistroviridae |
| SAL_4_Contig_98_20 | 20 | 152 | 1E^-19^ | 96 | 86 | Shuangao noda-like virus 1 | 0 | 0 | 0 | 20 | RNA | Unclassified |
| SAL_4_Contig_95_20 | 20 | 188 | 7E^-13^ | 90 | 56 | Macrobrachium rosenbergii nodavirus | 0 | 0 | 0 | 20 | ssRNA | Nodaviridae |
| SAL_4_Contig_45_20 | 20 | 265 | 7E^-32^ | 98 | 67 | Changjiang picorna-like virus 17 | 0 | 0 | 0 | 20 | RNA | Unclassified |
| SAL_1_Contig_138_20 | 20 | 266 | 4E^-40^ | 98 | 75 | SINV-1 | 20 | 0 | 0 | 0 | ssRNA | Dicistroviridae |
| SAL_1_Contig_134_20 | 20 | 270 | 3E^-50^ | 95 | 94 | Wuhan insect virus 11 | 20 | 0 | 0 | 0 | RNA | Unclassified |
| SAL_2_Contig_55_20 | 20 | 288 | 2E^-38^ | 96 | 71 | Hubei picorna-like virus 46 | 0 | 20 | 0 | 0 | RNA | Unclassified |
| SAL_2_Contig_80_20 | 20 | 295 | 1E^-45^ | 95 | 83 | Bat iflavirus | 0 | 20 | 0 | 0 | RNA | Unclassified |
| SAL_4_Contig_92_19 | 19 | 300 | 5E^-26^ | 99 | 52 | Nyamanini nyavirus | 0 | 0 | 0 | 19 | ssRNA | Nyamiviridae |
| SAL_3_Contig_66_18 | 18 | 133 | 2E^-10^ | 94 | 64 | Penguinpox virus | 0 | 0 | 18 | 0 | dsDNA | Poxviridae |
| SAL_3_Contig_82_18 | 18 | 228 | 1E^-38^ | 89 | 97 | Newington virus | 0 | 0 | 18 | 0 | ssRNA | Nodaviridae |
| SAL_4_Contig_97_18 | 18 | 230 | 1E^-31^ | 96 | 80 | Shuangao noda-like virus 1 | 0 | 0 | 0 | 18 | RNA | Unclassified |
| SAL_4_Contig_87_18 | 18 | 475 | 1E^-68^ | 98 | 65 | Shuangao insect virus 11 | 0 | 0 | 0 | 18 | RNA | Unclassified |
| SAL_2_Contig_26_17 | 17 | 184 | 3E^-31^ | 97 | 88 | Beihai picorna-like virus 76 | 0 | 17 | 0 | 0 | RNA | Unclassified |
| SAL_3_Contig_24_17 | 17 | 249 | 9E^-29^ | 100 | 62 | Sanxia water strider virus 9 | 0 | 0 | 17 | 0 | RNA | Unclassified |
| SAL_1234_Contig_11 | 17 | 305 | 1E^-41^ | 74 | 94 | Shuangao insect virus 8 | 17 | 0 | 0 | 0 | RNA | Unclassified |
| SAL_2_Contig_91_14 | 14 | 232 | 1E^-26^ | 76 | 83 | Hubei orthoptera virus 1 | 0 | 14 | 0 | 0 | RNA | Unclassified |
| SAL_3_Contig_56_13 | 13 | 230 | 8E^-06^ | 39 | 70 | Hubei noda-like virus 11 | 0 | 0 | 13 | 0 | RNA | Unclassified |
| SAL_4_Contig_23_12 | 12 | 213 | 2E^-38^ | 100 | 85 | Rhopalosiphum padi virus | 0 | 0 | 0 | 12 | ssRNA | Dicistroviridae |
| SAL_1_Contig_133_12 | 12 | 272 | 4E^-44^ | 99 | 79 | Hubei orthoptera virus 1 | 12 | 0 | 0 | 0 | RNA | Unclassified |
| SAL_1_Contig_38_12 | 12 | 441 | 8E^-64^ | 100 | 68 | Hubei picorna-like virus 41 | 12 | 0 | 0 | 0 | RNA | Unclassified |
| SAL_4_Contig_101_12 | 12 | 502 | 4E^-102^ | 99 | 86 | Solenopsis invicta densovirus | 0 | 0 | 0 | 12 | ssDNA | Parvoviridae |
| SAL_2_Contig_42_11 | 11 | 239 | 3E^-13^ | 94 | 46 | Wenzhou picorna-like virus 29 | 0 | 11 | 0 | 0 | RNA | Unclassified |
| SAL_1_Contig_139_11 | 11 | 263 | 2E^-50^ | 92 | 99 | Spodoptera exigua iflavirus 1 | 11 | 0 | 0 | 0 | ssRNA | Iflaviridae |
| SAL_4_Contig_61_11 | 11 | 295 | 3E^-22^ | 99 | 50 | Acute bee paralysis virus | 0 | 0 | 0 | 11 | ssRNA | Dicistroviridae |
| SAL_4_Contig_93_10 | 10 | 131 | 2E^-21^ | 96 | 88 | Shuangao noda-like virus 1 | 0 | 0 | 0 | 10 | RNA | Unclassified |
| SAL_4_Contig_104_10 | 10 | 139 | 2E^-17^ | 97 | 80 | Shuangao noda-like virus 1 | 0 | 0 | 0 | 10 | RNA | Unclassified |
| SAL_1_Contig_70_10 | 10 | 142 | 3E^-23^ | 99 | 98 | Alphanodavirus HB-2007 | 10 | 0 | 0 | 0 | ssRNA | Unclassified |
| SAL_4_Contig_88_10 | 10 | 152 | 4E^-15^ | 98 | 68 | Shuangao noda-like virus 1 | 0 | 0 | 0 | 10 | RNA | Unclassified |
| SAL_2_Contig_103_10 | 10 | 175 | 2E^-14^ | 96 | 61 | Shuangao insect virus 11 | 0 | 10 | 0 | 0 | RNA | Unclassified |
| SAL_1_Contig_170_10 | 10 | 189 | 7E^-16^ | 98 | 58 | Hubei orthoptera virus 1 | 10 | 0 | 0 | 0 | RNA | Unclassified |
| SAL_3_Contig_39_10 | 10 | 204 | 2E^-11^ | 97 | 53 | Aphis glycines virus 1 | 0 | 0 | 10 | 0 | RNA | Unclassified |
| SAL_2_Contig_30_10 | 10 | 218 | 5E^-28^ | 97 | 68 | Washington bat picornavirus | 0 | 10 | 0 | 0 | ssRNA | Picornaviridae |
| SAL_1_Contig_156_10 | 10 | 226 | 6E^-07^ | 84 | 43 | SINV-1 | 10 | 0 | 0 | 0 | ssRNA | Dicistroviridae |
| SAL_1_Contig_122_10 | 10 | 300 | 8E^-37^ | 98 | 68 | Drosophila C virus | 10 | 0 | 0 | 0 | ssRNA | Dicistroviridae |
| SAL_4_Contig_12_10 | 10 | 300 | 3E^-29^ | 96 | 60 | Bat badicivirus 1 | 0 | 0 | 0 | 10 | RNA | Unclassified |
| SAL_1_Contig_117_10 | 10 | 301 | 2E^-22^ | 98 | 45 | SINV-2 | 10 | 0 | 0 | 0 | ssRNA | Unclassified |
| SAL_3_Contig_8_10 | 10 | 301 | 3E^-56^ | 89 | 93 | Acute bee paralysis virus | 0 | 0 | 10 | 0 | ssRNA | Dicistroviridae |
| SAL_4_Contig_91_10 | 10 | 373 | 4E^-70^ | 98 | 85 | Solenopsis invicta densovirus | 0 | 0 | 0 | 10 | ssDNA | Parvoviridae |
| SAL_3_Contig_85_10 | 10 | 555 | 3E^-49^ | 98 | 49 | Wuhan arthropod virus 2 | 0 | 0 | 10 | 0 | RNA | Unclassified |
| SAL_3_Contig_72_9 | 9 | 170 | 2E^-20^ | 98 | 83 | Drosophila C virus | 0 | 0 | 9 | 0 | ssRNA | Dicistroviridae |
| SAL_1_Contig_62_9 | 9 | 176 | 1E^-15^ | 98 | 62 | Aphid lethal paralysis virus | 9 | 0 | 0 | 0 | ssRNA | Dicistroviridae |
| SAL_4_Contig_10_9 | 9 | 248 | 7E^-09^ | 87 | 38 | Hubei odonate virus 3 | 0 | 0 | 0 | 9 | RNA | Unclassified |
| SAL_4_Contig_102_9 | 9 | 265 | 2E^-09^ | 88 | 43 | Solenopsis invicta densovirus | 0 | 0 | 0 | 9 | ssDNA | Parvoviridae |
| SAL_4_Contig_148_9 | 9 | 309 | 2E^-56^ | 100 | 91 | Acute bee paralysis virus | 0 | 0 | 0 | 9 | ssRNA | Dicistroviridae |
| SAL_3_Contig_6_9 | 9 | 454 | 2E^-66^ | 99 | 69 | Hubei picorna-like virus | 0 | 0 | 9 | 0 | RNA | Unclassified |
| SAL_2_Contig_93_9 | 9 | 725 | 3E^-160^ | 98 | 93 | Aphid lethal paralysis virus | 0 | 9 | 0 | 0 | ssRNA | Dicistroviridae |
| SAL_4_Gp_N_Contig_6_8 | 8 | 122 | 2E^-11^ | 95 | 67 | Amsacta moorei entomopoxvirus | 0 | 0 | 0 | 8 | dsDNA | Poxviridae |
| SAL_4_Contig_56_8 | 8 | 205 | 3E^-23^ | 98 | 67 | Crab spider picornavirus | 0 | 0 | 0 | 8 | ssRNA | Unclassified |
| SAL_4_Contig_13_8 | 8 | 217 | 5E^-20^ | 91 | 62 | Hubei picorna-like virus 40 | 0 | 0 | 0 | 8 | RNA | Unclassified |
| SAL_3_Contig_49_8 | 8 | 227 | 1E^-10^ | 93 | 43 | Beihai sipunculid worm virus 5 | 0 | 0 | 8 | 0 | RNA | Unclassified |
| SAL_3_Contig_11_8 | 8 | 266 | 2E^-26^ | 85 | 61 | Wuhan arthropod virus 2 | 0 | 0 | 8 | 0 | RNA | Unclassified |
| SAL_1_Contig_121_8 | 8 | 300 | 1E^-31^ | 99 | 61 | Washington bat picornavirus | 8 | 0 | 0 | 0 | ssRNA | Picornaviridae |
| SAL_2_Contig_101_8 | 8 | 303 | 1E^-41^ | 98 | 74 | Hubei picorna-like virus 23 | 0 | 8 | 0 | 0 | RNA | Unclassified |
| SAL_2_Contig_81_8 | 8 | 391 | 6E^-39^ | 98 | 89 | Hubei picorna-like virus 23 | 0 | 8 | 0 | 0 | RNA | Unclassified |
| SAL_1_Contig_179_7 | 7 | 175 | 9E^-32^ | 99 | 93 | Acute bee paralysis virus | 7 | 0 | 0 | 0 | ssRNA | Dicistroviridae |
| SAL_1_Contig_135_7 | 7 | 269 | 3E^-12^ | 98 | 42 | Shuangao insect virus 8 | 7 | 0 | 0 | 0 | RNA | Unclassified |
| SAL_1_Contig_25_7 | 7 | 395 | 6E^-20^ | 82 | 46 | Hubei picorna-like virus 15 | 7 | 0 | 0 | 0 | RNA | Unclassified |
| SAL_4_Contig_103_7 | 7 | 417 | 6E^-19^ | 79 | 45 | Solenopsis invicta densovirus | 0 | 0 | 0 | 7 | ssDNA | Parvoviridae |
| SAL_2_Contig_8_7 | 7 | 549 | 6E^-91^ | 99 | 83 | Shuangao insect virus 8 | 0 | 7 | 0 | 0 | RNA | Unclassified |
| SAL_1_Contig_69_6 | 6 | 142 | 2E^-13^ | 99 | 64 | Hubei picorna-like virus 48 | 6 | 0 | 0 | 0 | RNA | Unclassified |
| SAL_3_Contig_74_6 | 6 | 155 | 4E^-15^ | 92 | 67 | Hubei orthoptera virus 1 | 0 | 0 | 6 | 0 | RNA | Unclassified |
| SAL_3_Contig_65_6 | 6 | 159 | 8E^-11^ | 90 | 65 | Burke-Gilman virus | 0 | 0 | 6 | 0 | RNA | Unclassified |
| SAL_1_Contig_178_6 | 6 | 177 | 8E^-30^ | 98 | 97 | Alphanodavirus HB-2007 | 6 | 0 | 0 | 0 | ssRNA | Unclassified |
| SAL_1_Contig_173_6 | 6 | 183 | 5E^-27^ | 100 | 77 | SINV-1 | 6 | 0 | 0 | 0 | ssRNA | Dicistroviridae |
| SAL_2_Contig_67_6 | 6 | 217 | 4E^-39^ | 99 | 94 | Wuhan insect virus 33 | 0 | 6 | 0 | 0 | RNA | Unclassified |
| SAL_1_Contig_151_6 | 6 | 237 | 1E^-21^ | 93 | 57 | Beihai sipunculid worm virus 5 | 6 | 0 | 0 | 0 | RNA | Unclassified |
| SAL_1_Contig_150_6 | 6 | 243 | 1E^-47^ | 100 | 100 | Himetobi P virus | 6 | 0 | 0 | 0 | ssRNA | Dicistroviridae |
| SAL_4_Contig_96_6 | 6 | 324 | 2E^-19^ | 90 | 47 | Solenopsis invicta densovirus | 0 | 0 | 0 | 6 | ssDNA | Parvoviridae |
| SAL_4_Contig_145_6 | 6 | 353 | 1E^-65^ | 99 | 91 | Acute bee paralysis virus | 0 | 0 | 0 | 6 | ssRNA | Dicistroviridae |
| SAL_4_Gp_M_Contig_70_6 | 6 | 603 | 3E^-65^ | 90 | 58 | Spodoptera exigua virus AKJ-2014 | 0 | 0 | 0 | 6 | ssRNA | Picornaviridae |
| SAL_2_Gp_A_Contig_11_5 | 5 | 217 | 9E^-12^ | 64 | 62 | Formica exsecta virus 1 | 0 | 5 | 0 | 0 | ssRNA | Unclassified |
| SAL_3_Contig_14_5 | 5 | 239 | 6E^-10^ | 95 | 35 | Hubei picorna-like virus 78 | 0 | 0 | 5 | 0 | RNA | Unclassified |
| SAL_3_Contig_44_5 | 5 | 300 | 2E^-24^ | 97 | 54 | Helicoverpa armigera iflavirus | 0 | 0 | 5 | 0 | ssRNA | Unclassified |
| SAL_4_Contig_20_5 | 5 | 300 | 9E^-34^ | 99 | 58 | Beihai picorna-like virus 77 | 0 | 0 | 0 | 5 | RNA | Unclassified |
| SAL_1234_Contig_44 | 4 | 188 | 7E^-19^ | 65 | 95 | SINV-1 | 0 | 0 | 4 | 0 | ssRNA | Dicistroviridae |
| SAL_4_Contig_79_8 | 4 | 233 | 5E^-31^ | 99 | 60 | Orinoco virus | 0 | 0 | 0 | 4 | RNA | Unclassified |
| SAL_2_Contig_66_4 | 4 | 300 | 9E^-37^ | 99 | 67 | Homalodisca coagulata virus 1 | 0 | 4 | 0 | 0 | ssRNA | Dicistroviridae |
| SAL_3_Contig_4_4 | 4 | 300 | 4E^-41^ | 98 | 69 | Aphis glycines virus 1 | 0 | 0 | 4 | 0 | RNA | Unclassified |
| SAL_2_Contig_109_4 | 4 | 311 | 2E^-63^ | 99 | 99 | Aphid lethal paralysis virus | 0 | 4 | 0 | 0 | ssRNA | Dicistroviridae |
| SAL_2_Contig_115_4 | 4 | 329 | 4E^-14^ | 89 | 40 | Wenzhou picorna-like virus 37 | 0 | 4 | 0 | 0 | RNA | Unclassified |
| SAL_4_Gp_I_Contig_35_4 | 4 | 329 | 2E^-58^ | 99 | 83 | Drosophila C virus | 0 | 0 | 0 | 4 | ssRNA | Dicistroviridae |
| SAL_4_Gp_D_Contig_55_4 | 4 | 356 | 4E^-68^ | 98 | 93 | Acute bee paralysis virus | 0 | 0 | 0 | 4 | ssRNA | Dicistroviridae |
| SAL_2_Gp_A_Contig_42_4 | 4 | 474 | 6E^-27^ | 99 | 42 | Israeli acute paralysis virus | 0 | 4 | 0 | 0 | ssRNA | Dicistroviridae |
| SAL_2_Contig_61_4 | 4 | 488 | 2E^-59^ | 95 | 64 | Hubei orthoptera virus 1 | 0 | 4 | 0 | 0 | RNA | Unclassified |
| SAL_2_Contig_5_3 | 3 | 301 | 1E^-12^ | 93 | 40 | Hubei picorna-like virus 15 | 0 | 3 | 0 | 0 | RNA | Unclassified |
| SAL_2_Contig_113_3 | 3 | 338 | 6E^-73^ | 99 | 98 | Black queen cell virus | 0 | 3 | 0 | 0 | ssRNA | Dicistroviridae |
| SAL_1234_Contig_1 | 3 | 396 | 2E^-43^ | 57 | 97 | Solenopsis invicta virus 1A | 0 | 0 | 0 | 3 | ssRNA | Dicistroviridae |
| SAL_1_Contig_119_2 | 2 | 300 | 1E^-11^ | 95 | 35 | Hubei picorna-like virus 15 | 2 | 0 | 0 | 0 | RNA | Unclassified |
| SAL_2_Contig_11_2 | 2 | 300 | 3E^-13^ | 96 | 39 | Israeli acute paralysis virus | 0 | 2 | 0 | 0 | ssRNA | Dicistroviridae |
| SAL_4_Contig_31_2 | 2 | 308 | 2E^-07^ | 62 | 41 | Beihai narna-like virus 18 | 0 | 0 | 0 | 2 | RNA | Unclassified |
| SAL_1_Contig_115_2 | 2 | 370 | 1E^-08^ | 68 | 36 | Aphid lethal paralysis virus | 2 | 0 | 0 | 0 | ssRNA | Dicistroviridae |
| SAL_1_Contig_18_2 | 2 | 478 | 1E^-31^ | 97 | 46 | Hubei picorna-like virus 15 | 2 | 0 | 0 | 0 | RNA | Unclassified |
| SAL_3_MB00268:70:Singleton_ABH2W:1:2114:19243:10671_12296 | 1 | 300 | 2E^-33^ | 81 | 72 | Hubei orthoptera virus 1 | 0 | 0 | 1 | 0 | RNA | Unclassified |
| SAL_4_MA00268:70:Singleton_ABH2W:1:1101:8653:8354_317 | 1 | 300 | 8E^-18^ | 54 | 72 | Wuhan insect virus 11 | 0 | 0 | 0 | 1 | RNA | Unclassified |
| SAL_4_MA00268:70:Singleton_ABH2W:1:2101:22687:12397_8732 | 1 | 300 | 4E^-22^ | 97 | 47 | Israeli acute paralysis virus | 0 | 0 | 0 | 1 | ssRNA | Dicistroviridae |
| SAL_3_ME00268:70:Singleton_ABH2W:1:1106:3727:18273_42826 | 1 | 300 | 7E^-14^ | 99 | 39 | Rolda virus | 0 | 0 | 1 | 0 | RNA | Unclassified |
| SAL_4_MA00268:70:Singleton_ABH2W:1:1117:10103:14268_7265 | 1 | 301 | 3E^-47^ | 88 | 84 | Kasmir bee virus | 0 | 0 | 0 | 1 | ssRNA | Dicistroviridae |
